# Does concurrent breastfeeding alongside the introduction of solid food prevent the development of food allergy?

**DOI:** 10.1017/jns.2016.31

**Published:** 2016-10-03

**Authors:** Carina Venter, Kate Maslin, Taraneh Dean, Syed Hasan Arshad

**Affiliations:** 1 School of Health Sciences and Social Work, University of Portsmouth, Portsmouth, UK; 2 David Hide Asthma and Allergy Research Centre, Newport, Isle of Wight, UK; 3 Clinical and Experimental Sciences, University of Southampton, Southampton, UK

**Keywords:** Food allergies, Infant feeding, Breastfeeding, Complementary feeding, CMA, cows’ milk allergy, EAT, Enquiring About Tolerance, ESPGHAN, European Society of Paediatric Gastroenterology, Hepatology and Nutrition, FAIR, Food Allergy and Intolerance Research, LEAP, Learning Early About Peanut Allergy

## Abstract

The timing of introduction of solid food on the subsequent development of food allergy is under debate and the role of concurrent breastfeeding is unclear. The aim of the present study was to investigate the role of solid food introduction whilst concurrently breastfeeding on food allergy outcome, with a specific focus on cows’ milk allergy. Prospectively collected infant feeding data from a birth cohort were analysed. Participants with histories suggestive of food allergy underwent diagnostic food challenges. Children with food allergy were matched to control participants for age and sex. Mann–Whitney *U* tests, χ^2^, Fisher exact tests and logistic regression calculations were undertaken. A total of thirty-nine food-allergic children and seventy-eight matched controls were identified, including twenty-two cows’ milk-allergic children and forty-four matched controls. The control group introduced solid food earlier than the food-allergic group (*P* < 0·05). There was no effect of concurrent breastfeeding alongside cows’ milk introduction or other food allergens on the development of food allergy. Due to small numbers, it was not possible to explore differences for food allergy phenotype. We have therefore found no evidence that introducing solids, or food allergens, whilst breastfeeding has an allergy-preventative effect; however, the results should be interpreted with caution due to sample size. Recommendations regarding infant feeding and food allergy should be carefully considered. Although breastfeeding should be promoted for many health reasons, larger studies looking at the introduction of food allergens on the development of food allergy are needed to make a final conclusion.

The introduction of solid food to infants’ diets is a transitional phase from a solely milk-based diet to family meals, allowing infants to meet their nutritional requirements in line with developmental needs. The WHO recommends that infants are exclusively breastfed until 6 months of age, followed by introduction of solid food at 6 months^(^^1^^)^. In the UK, the Department of Health adapted this advice slightly, recommending that introduction of solid food should take place at around 6 months, with the caveat that solid foods should never be given to babies under 17 weeks old^(^^2^^)^.

A number of studies investigating the effect of food allergen introduction during breastfeeding have been published^(^^3^^–^^9^^)^ with inconsistent findings. A large population birth cohort study in Israel has suggested that very early exposure to cows’ milk, in the form of infant formula in combination with breastfeeding, might protect against IgE-mediated cows’ milk allergy (CMA)^(^^10^^)^, which is clearly in contradiction to WHO advice about exclusive breastfeeding. With the recent publication of the Learning Early About Peanut Allergy (LEAP) study^(^^7^^)^ data reporting that early introduction of peanut to high-risk infants is protective against peanut allergy and the subsequent publication of an international expert opinion consensus document^(^^11^^)^, the question about the introduction of allergenic foods while breastfeeding has become even more crucial to answer.

The literature regarding early feeding and the development of coeliac disease and wheat allergy illustrates the lack of consensus. In 2002, Ivarsson *et al*.^(^^3^^)^ published data comparing 627 cases with coeliac disease and 1254 controls, reporting that introducing gluten whilst breastfeeding seemed to have a protective effect against coeliac disease. This recommendation was included in the European Society of Paediatric Gastroenterology, Hepatology and Nutrition (ESPGHAN) commentary^(^^12^^)^ on complementary feeding. This was followed by the publication from a Swedish birth cohort, by Ivarsson *et al*.^(^^4^^)^ in 2013, finding that gradual introduction of wheat- or gluten-containing foods from 4 months of age, whilst breast feeding, may prevent the development of coeliac disease – although this was disputed in a subsequent meta-analysis^(^^5^^)^ with Ivarsson as one of the co-authors. The most recent ESPGHAN position paper states that gluten may be introduced into the infant's diet any time between 4 and 12 months of age (irrespective of being breastfed or not), although optimal amounts of gluten to be introduced are not known^(^^13^^)^.

Looking specifically at data from the UK, Grimshaw *et al*.^(^^9^^)^ reported that based on a nested case–control study of forty-one food-allergic and eighty-two control participants (including twenty infants with CMA and forty-one control infants), there was no statistically significant difference between the food-allergic and non-food-allergic group in terms of introduction of any form of cows’ milk into the diet. However, introduction of cows’ milk-containing foods or drinks while concurrently breastfeeding seemed to have a protective effect against the development of overall food allergy by 2 years of age^(^^9^^)^. More recent data from the same cohort have shown that ‘concurrent breastfeeding with cows’ milk from any source’ was a risk factor for non-IgE-mediated food allergy, but not for IgE-mediated food allergy^(^^8^^)^. The aim of the present study therefore was to analyse our dataset from the Food Allergy and Intolerance Research (FAIR)^(^^14^^)^ cohort to investigate cows’ milk introduction and concurrent breastfeeding on food allergy outcome, with a particular emphasis on CMA outcome.

## Methods

In brief, the FAIR birth cohort born on the Isle of Wight (UK)^(^^15^^)^ between 2001 and 2002 was followed up prospectively (*n* 969). All pregnant mothers with an estimated delivery date of September 2001 to August 2002 were approached at antenatal clinics. The cohort was predominantly Caucasian. The majority of mothers were of medium-high socio-economic status (60 %), with 41·4 % being primiparous. Information was obtained at 12 weeks’ gestation, at the date of delivery and prospectively during the first year of life at 3, 6, 9 and 12 months. Children were clinically examined and skin prick tested to a panel of key food allergens (ALK Abello) (namely, milk, egg, wheat, cod and peanut) at 1 year of age and invited for food challenges when indicated. The Committee on Toxicity advice (UK)^(^^16^^)^, which recommended the avoidance of peanuts until 3 years of age in high-risk families, was still relevant at the time. Children were therefore first challenged to peanuts at 3 years of age. Children were challenged to 10 g of dried food as per protocols previously described^(^^15^^)^. For CMA, children were challenged to 10 g of milk powder. Challenges for the diagnosis of delayed-type CMA (non-IgE CMA) were performed over a 1-week period, giving a normal portion of cows’ milk per d^(^^17^^)^. All challenges were performed as double-blind placebo-controlled food challenges where possible.

Introduction of milk was defined as introduction of infant formula containing milk (specified in days) and any food or drink containing milk at 3–6 months, 6–9 months, 9–12 months and >12 months. Wheat introduction referred to wheat-containing food (e.g. wheat-based breakfast cereal), egg only referred to whole egg introduction according to the set categories and peanut introduction referred to any peanut or peanut-containing foods according to set categories. This was different from our publication in 2009^(^^18^^)^, where we defined exposure to an allergen by also taking maternal intake during breastfeeding into account. Ethical approval for the study was obtained from the Isle of Wight, Portsmouth and South East Hampshire Local Research Ethics Committee (reference 09/01). Written consent was obtained from parents of participants.

### Statistical analysis

#### Power calculation

Using a 2:1 ratio study design with 80 % power and 95 % CI would require ninety-six CMA participants with 192 control participants to detect a difference in concurrent breastfeeding and cows’ milk consumption in the development of food allergy.

#### Data analysis

Food-allergic children were matched to two children of the same sex recruited consecutively to them. As mothers were recruited from antenatal clinics consecutively throughout the year from September 2001 to August 2002, this means that consecutive children listed in the database were usually the next closest in age. Data were double entered and verified and statistical analysis was conducted by using SPSS version 21 (IBM SPSS Statistics, IBM Corp.). Continuous variables were described by medians and ranges, as data were not normally distributed. Categorical variables were described in terms of numbers and percentages. Descriptive statistics, χ^2^ tests, Fisher exact tests and Mann–Whitney *U* tests were used to compare the CMA group with the control group. A conditional logistic regression model was used to determine factors predicting CMA.

## Results

**Fig. 1.**
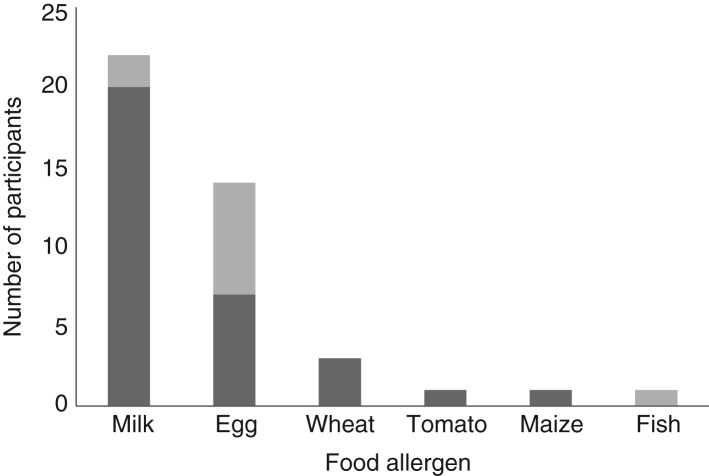
Participant food allergies. 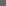, Non-IgE-mediated allergy; 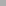, IgE-mediated allergy.

A total of thirty-nine food-allergic children and seventy-eight matched controls were identified from the dataset. Of the children, eleven had IgE-mediated food allergy and twenty-eight had non-IgE-mediated food allergy. The thirty-nine food-allergic children and seventy-eight matched controls included twenty-two children with CMA and forty-four matched controls. Of the children, two had IgE CMA, whilst twenty had non-IgE CMA. In addition to cows’ milk, participants in the dataset of food-allergic children (*n* 39) had allergies to egg, wheat, tomato, maize and fish (shown in Fig. 1).

### Data on children with food allergy compared with matched controls

**Fig. 2.**
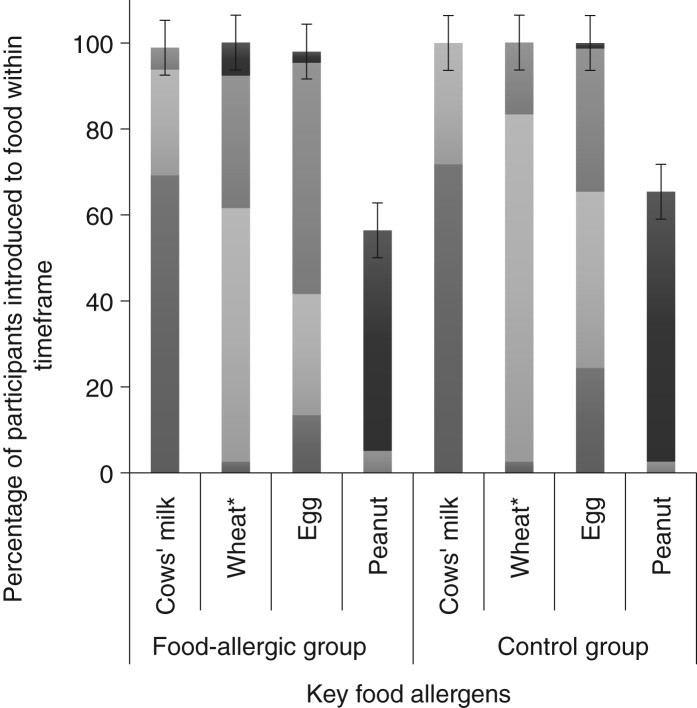
Timing of introduction of key food allergens to food-allergic (*n* 39) and control groups (*n* 78). * Significantly different between groups using Fisher's exact test (*P* = 0·011). 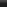, 9–12 months; 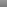, 6–9 months; 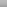, 3–6 months; 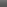, < 3 months. The error bars indicate 95 % confidence intervals.

Tables 1 and 2 show demographic and infant feeding characteristics of participants. Comparing the food-allergic group with the control group, there was no difference for any sociodemographic variable. We found no difference in terms of the prevalence of cows’ milk intake concurrently with breastfeeding (56·4 % in the food-allergic group and 55·1 % in the control group; *P* = 0·90) (Table 1). The only differences we found between the two groups were age of introduction of wheat (*P* = 0·011) and age of introduction of solid food (*P* = 0·037). In both cases the control group introduced wheat and solid food earlier. The timing of introduction of key food allergens is shown in Fig. 2. There was no difference between groups for duration of concurrent breastfeeding with consumption of all solids or any key food allergen (data not shown).

**Table 1.** Demographic and infant feeding characteristics of children with food allergy and matched controls (Numbers of participants and percentages; medians and ranges)

	Food allergy group (*n* 39)		Control group (*n* 78)		
Characteristics	*n*	%	*n*	%	*P*
Male	25	64	51	65	0·98
Birth weight (kg)					0·15
Median	3·8		3·6		
Range	2·09–4·54		2·13–4·80		
Maternal age (years)					0·56
Median	29		29·5		
Range	20–44		18–43		
Maternal education					
School	10	25·6	21	26·9	0·21
Further	15	38·5	40	51·3	
Higher	14	35·9	15	19·2	
Did not complete school	0	0	2	2·6	
Maternal smoking	6	15·4	16	20·5	0·50
Only child	21	53·8	27	34·6	0·58
Maternal asthma	9	23·1	18	23·1	1·00
Maternal hayfever	16	41·0	26	33·3	0·41
Maternal eczema	13	33·3	19	24·4	0·31
Maternal food allergy	12	30·8	13	16·7	0·43
Initiation of breastfeeding	31	79·5	55	70·5	0·30
Breastfeeding duration (d)					0·58
Median	56		42		
Range	0–730		0–730		
Exclusive breastfeeding duration (d)					0·74
Median	45		21		
Range	0–182		0–168		
Solid food introduction (d)					0·037*
Median	112		112		
Range	84–168		42–168		
Bottle feeding introduced (d)					0·38
Median	31·5		11		
Range	0–276		0–276		
Any concurrent breastfeeding and cows’ milk consumption	22	56·4	43	55·1	0·90

* *P* < 0·05.

**Table 2.** Demographic and infant feeding characteristics of children with cows’ milk allergy and matched controls (Numbers of participants and percentages; medians and ranges)

	Cows’ milk allergy group (*n* 22)		Control group (*n* 44)		
Characteristics	*n*	%	*n*	%	*P*
Male	16	73	33	75	0·84
Birth weight (kg)					0·13
Median	3·4		3·6		
Range	2·09–4·54		2·66–4·80		
Maternal age (years)					0·55
Median	28·5		29·5		
Range	21–44		19–43		
Maternal education					
School	6	27	10	22·7	0·09
Further	8	36·5	27	61·4	
Higher	8	36·5	6	13·6	
Did not complete school	0	0	1	2·3	
Maternal smoking	4	18·2	9	20·5	0·83
Only child	9	40·9	14	31·8	0·58
Maternal asthma	6	27	10	22·7	0·69
Maternal hayfever	10	45·5	16	36·4	0·48
Maternal eczema	8	36·5	11	25	0·34
Maternal food allergy	7	31·8	8	18·2	0·21
Initiation of breastfeeding	15	68·2	32	72·7	0·70
Breastfeeding duration (d)					0·44
Median	15		49		
Range	0–730		0–730		
Exclusive breastfeeding duration (d)					0·29
Median	2		24·5		
Range	0–140		0–168		
Age of solid food introduction (d)					0·014*
Median	112		112		
Range	84–168		42–168		
Bottle feeding introduced (d)					0·12
Median	1		14		
Range	0–126		0–259		
Any concurrent breastfeeding and cows’ milk consumption	9	40·9	26	59·1	0·16

* *P* < 0·05.

### Data on children with cows’ milk allergy compared with matched controls

**Fig. 3.**
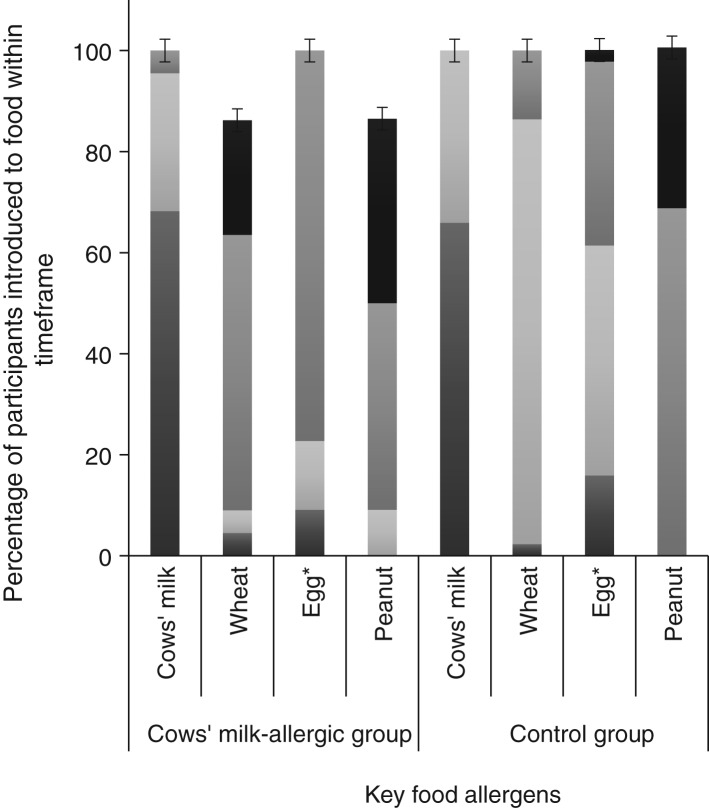
Timing of introduction of key food allergens to cows’ milk-allergic (*n* 22) and control groups (*n* 44). *Significantly different between groups using Fisher's exact test (*P* = 0·012). 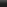, 9–12 months; 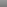, 6–9 months; 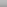, 3–6 months; 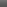, < 3 months. The error bars indicate 95 % confidence intervals.

Comparing the group of CMA infants (*n* 22) with the matched controls (*n* 44), there was no difference between groups for any sociodemographic variables (Table 2). We found statistically significant differences for age of introduction of solid foods (*P* = 0·014), in addition to age of introduction of egg (*P* = 0·012), but no difference for the other three key food allergens. Timing of introduction of key food allergens is shown in Fig. 3. A greater proportion of the control group was introduced to egg and solids earlier than the CMA group. Although the median age of introduction of solids was the same for both groups (112 d), the range of data shows the control group introduced solids earlier (42 *v*. 84 d) (Table 2).

There was no difference in the prevalence of cows' milk introduction concurrently with breast feeding (40·9 % in the CMA and 59·1 % in the control group; *P* = 0·16). There was no significant difference between the CMA and control group for duration of concurrent breastfeeding and consumption of all solids or any key food allergen. As the data were skewed, analyses were repeated excluding infants who were not breastfed at all, again finding no significant difference for concurrent breastfeeding and consumption of all solids, or any key food allergen. The variables were recoded to provide a categorical ‘concurrent breastfeeding’ variable for each of the key food allergens, in order to determine if there was a difference between CMA and control groups for any duration of concurrent breastfeeding with any key food allergen. There was no significant difference between the groups.

The initial conditional logistical regression model for prediction of CMA contained five independent variables, based on variables that were shown to be statistically different between groups (age of solid introduction) and factors that are thought to be associated with food allergy development identified in previous literature (duration of concurrent breastfeeding and cows’ milk consumption, breastfeeding duration in weeks, maternal eczema and maternal food allergy). This model did not significantly predict CMA, therefore duration of concurrent breastfeeding and cows’ milk consumption and maternal eczema were removed in a backward elimination stepwise fashion, leaving a significant model containing three variables (χ^2^ (3, *n* 66) = 9·698; *P* = 0·037). Only one of the variables, age of solid food introduction (*P* = 0·011), made a unique statistically significant contribution to the model, shown in Table 3.

**Table 3.** Conditional logistic regression model for assessment of variables associated with development of cows’ milk allergy (Adjusted odds ratios and 95 % confidence intervals)

	Adjusted odds ratio	95 % CI
Breastfeeding duration (d)	0·990	0·978, 1·001
Age at solid food introduction (d)	1·024*	1·005, 1·043
Maternal food allergy	0·730	0·730, 1·828

* *P* < 0·05.

## Discussion

The present study aimed to show whether breastfeeding alongside solid food introduction influences the development of food allergy, with an emphasis on CMA. We compared a group of children with food allergy at the age of 1 year with matched controls, specifically focusing on the infants with CMA. We found no difference between food-allergic and control groups, other than earlier age of introduction of solid foods and earlier introduction of wheat in the control group. Age of introduction of solid food was the only significant independent contributor to the regression model in prediction of CMA. Our data indicate, in contrast to Grimshaw *et al*.^(^^8^^,^^9^^)^ and Poole *et al*.^(^^5^^)^, that introduction of food allergens whilst concurrently breastfeeding does not prevent or increase the risk of the development of food allergies or CMA *per se*. This is particularly interesting as the FAIR birth cohort and the cohort studied by Grimshaw *et al*.^(^^9^^)^ were born approximately 5 years apart and only 20 miles (32 km) away from each other. Our ratio of non-IgE- to IgE-mediated CMA is very similar to those reported by the EuroPrevall sites in the UK and the Netherlands^(^^19^^)^. Due to small numbers, we did not, however, consider differences between children with IgE- and non-IgE-mediated food allergy, which may be an important factor to consider. A further difference is that we measured food allergy at age 1 year, whereas Grimshaw *et al*.^(^^8^^,^^9^^)^ reported food allergy data collected up to 2 years of age.

Although food allergy and coeliac disease are distinct entities, with differences in pathophysiology and natural history, parallels can be drawn by comparing prevention studies. We have learned from research looking at coeliac disease and introduction of gluten that although initial studies may indicate concurrent breastfeeding is protective, a later systematic review and study with a larger sample size have questioned this^(^^4^^,^^20^^)^. More recent research, including two randomised controlled trials of high-risk infants, has shown that exclusive breastfeeding and timing of introduction of gluten did not affect the development of coeliac disease^(^^21^^,^^22^^)^. The most recent ESPGHAN guidelines regarding the prevention of coeliac disease specify that in high-risk infants, earlier introduction of gluten is associated with earlier coeliac disease^(^^13^^)^.

Looking specifically at research of CMA, a prospective study of >13 000 infants in Israel^(^^10^^)^ reported that infants whose regular exposure to cows’ milk was withheld until the age of 4–6 months were at highest risk for IgE-mediated CMA, and those exposed to cows’ milk protein (in the form of infant formula) in the first 2 weeks of life had an extremely low incidence of IgE-mediated CMA. The recently published Enquiring About Tolerance (EAT) study^(^^6^^)^ was a randomised trial of introduction of allergenic foods in breastfed infants in the UK (*n* 1303). Exclusively breastfed infants were randomly assigned at 3 months of age to either: (i) the sequential early introduction of six allergenic foods (cows’ milk, peanut, egg, sesame, cod and wheat) between 3–6 months; or (ii) to a standard introduction group, who introduced allergenic foods from 6 months. The results indicated that in infants who adhered to the study protocol, the prevalence of any IgE-mediated food allergy was significantly lower in the early introduction group, than the standard introduction group. Specifically the prevalences of egg and peanut allergy were lower, but there was no difference with regard to milk, sesame, fish or wheat, which could perhaps be explained by poorer adherence to these foods. However, it is worth highlighting that the EAT study's endpoint was IgE-mediated allergy and it is known that the majority of CMA in the UK is non-IgE mediated^(^^14^^,^^19^^)^. Another recently published study, the Canadian Healthy Infant Longitudinal Development (CHILD) birth cohort study, has demonstrated that exclusive breastfeeding to 6 months does not significantly alter the risk of sensitisation to egg or peanut at age 1 year, but did increase the risk of sensitisation to cows’ milk^(^^23^^)^. However, the CHILD study was an observational study rather than an experimental study, using sensitisation rather than diagnosed food allergy as an end point and as yet has not published data on the role of concurrent food introduction during breastfeeding.

The European Academy of Allergy and Clinical Immunology^(^^24^^)^ recommends that ‘for all infants, exclusive breastfeeding is recommended for at least first 4–6 months of life’, which is equivalent to the UK Department of Health guidance^(^^2^^)^. In the USA, the Academy of Nutrition and Dietetics recommends a period of 6 months of exclusive breastfeeding^(^^25^^)^. However, the American Academy of Pediatrics^(^^26^^)^ and the American Academy of Asthma Allergy and Immunology^(^^27^^)^ recommend a 4- to 6-month period of exclusive breastfeeding for allergy prevention. Canadian allergy prevention guidelines emphasise that the total duration of breastfeeding (at least 6 months) may be more protective than exclusive breastfeeding for 6 months. The same guidelines also emphasise the importance of regular consumption (several times per week) once foods are introduced, in order to maintain tolerance^(^^28^^,^^29^^)^. Of note, the LEAP study protocol required children to have regular exposure to peanuts consuming 6 g per week eaten as 2 g of peanut protein consumed three times per week^(^^7^^)^. Following on from the LEAP^(^^7^^)^ study and the consensus statement on the early introduction of peanuts^(^^11^^)^, the National Institute of Allergy and Infectious Diseases has assembled a panel of experts from both the USA and Europe to review weaning and breastfeeding guidelines for allergy prevention. Although the LEAP study was limited to infants at high risk of peanut allergy, the EAT study results provide additional evidence using a cohort of infants from the general population^(^^6^^)^. It is therefore clear that complementary feeding and breastfeeding guidance may change. The debate is how this guidance will change in terms of introduction of allergens and what role concurrent breastfeeding whilst introducing food allergens (or any solid food) will play in reconstructing the guidelines.

Looking more broadly at allergic disease in general, rather than food allergy, we have also recently shown using two cohorts born on the Isle of Wight that the protective effects of non-exclusive and exclusive breastfeeding against long-term allergic outcomes were inconsistent, agreeing with previous observations of heterogeneous effects. Although breastfeeding should be recommended for other health benefits, following breastfeeding guidelines did not appear to afford consistent protection against long-term asthma, eczema, rhinitis or atopy. Further research is needed into the long-term effects of breastfeeding on allergic disease^(^^30^^)^.

There are of course many physiological and psychological reasons why mothers should be encouraged to breastfeed their infants^(^^1^^)^. However, we feel that more research is required regarding breastfeeding and allergy prevention, specifically around the topics of adherence and dose of food allergen exposure. In the UK, national data report that only 43 % of mothers are breastfeeding at 3 months and only 2/2700 children are exclusively breastfed at 6 months^(^^31^^)^. Initiation and continuation of breastfeeding are known to be emotive issues, with cessation and continuation both associated with feelings of guilt and shame^(^^32^^)^. It is known that several factors influence maternal decision making around infant feeding and that confusion exists around official guidelines^(^^33^^–^^35^^)^. Further advice regarding the introduction of allergens or solid foods whilst breastfeeding could pose an additional level of stress in mothers trying to prevent the development of allergic disease in their infant. Any revision of guidelines needs to take this into account, especially important when adherence to existing guidelines is already suboptimal. Although the EAT study protocol showed that earlier introduction of allergenic foods did not affect breastfeeding, it also demonstrated that achievement of consumption targets was only met by 42 % of those in the early introduction group. Consumption of the recommended amount and frequency of egg and sesame was particularly difficult^(^^36^^)^. Feeding guidelines should also take into consideration the role of developmental readiness to feed and responding to infants’ satiety cues. Nutritional guidelines that focus exclusively on food, rather than consider the feeding context, may unintentionally lead to controlling parental behaviours^(^^37^^)^.

The main limitation of our study is the small number of participants with CMA (*n* 22) and the small sample size of food-allergic participants overall (*n* 39). Due to small numbers it is not possible to conduct further meaningful subanalyses according to food allergy mechanism (IgE compared with non-IgE). The small sample size means that some statistical analyses may be underpowered with the risk of type II error (false negative) and indicates the need for larger studies. We have presented data regarding the introduction of food allergens within a 3-month period of time rather than quantity and frequency of exposure, as data were collected about timing of introduction, rather than dose. As with any observational study of infant feeding, it is not ethical to randomise or control the period of breastfeeding duration. Strengths of the study are the prospective research design, the age and sex matching of participants and the use of food challenges to diagnose food allergy.

In conclusion, we have found no evidence to suggest that introducing solids, or specifically key food allergens, whilst breastfeeding has an allergy-preventative effect; however, these data need to be interpreted with caution due to the small sample size of food- and milk-allergic children within the dataset. Breastfeeding rates in the UK and USA are suboptimal and there is confusion regarding infant feeding guidelines. Recommendations regarding infant feeding and food allergy should therefore be made with a considered approach. Although breastfeeding should of course be promoted for multiple nutritional, psychological and immunological reasons, large-scale studies examining the effects of timing of introduction of various food allergens on the development of IgE- and non-IgE-mediated food allergy, particularly in relation to breast feeding, are required before final conclusions can be drawn.
